# Thrombosis and Bleeding Risk Scores Are Strongly Associated with Mortality in Hospitalized Patients with COVID-19: A Multicenter Cohort Study

**DOI:** 10.3390/jcm13051437

**Published:** 2024-03-01

**Authors:** Kunapa Iam-Arunthai, Supat Chamnanchanunt, Pravinwan Thungthong, Poj Intalapaporn, Chajchawan Nakhahes, Tawatchai Suwanban, Ponlapat Rojnuckarin

**Affiliations:** 1 Division of Hematology, Department of Medicine, Rajavithi Hospital, College of Medicine, Rangsit University, Bangkok 10400, Thailand; 2 Department of Clinical Tropical Medicine, Faculty of Tropical Medicine, Mahidol University, Bangkok 10400, Thailand; 3 Division of Infectious Diseases, Department of Medicine, Rajavithi Hospital, College of Medicine, Rangsit University, Bangkok 10400, Thailand; 4 Center of Excellence in Translational Hematology, Department of Medicine, Faculty of Medicine, Chulalongkorn University and King Chulalongkorn Memorial Hospital, Bangkok 10330, Thailand

**Keywords:** COVID-19, Padua prediction score, IMPROVE score, venous thromboembolism, mortality

## Abstract

**Background:** Internationally established guidelines mention pharmacological prophylaxis for all hospitalized COVID-19 patients. However, there are concerns regarding the efficacy and safety of anticoagulants. This study investigated the associations between thrombosis/bleeding risk scores and clinical outcomes. **Methods:** We conducted a retrospective review of adult patients admitted to two hospitals between 2021 and 2022. We analyzed clinical data, laboratory results, low molecular weight heparin (LMWH) use, thrombosis, bleeding, and 30-day survival. **Results:** Of the 160 patients, 69.4% were female, and the median age was 59 years. The rates of thrombotic complications and mortality were 12.5% and 36.3%, respectively. LMWH prophylaxis was administered to 73 of the patients (45.6%). The patients with high Padua prediction scores (PPS) and high IMPROVE_VTE_ scores had a significantly higher risk of venous thromboembolism (VTE) compared to those with low scores (30.8% vs. 9.0%, *p* = 0.006 and 25.6% vs. 7.7%, *p* = 0.006). Similarly, elevated IMPROVE_VTE_ and IMPROVE_BRS_ scores were associated with increased mortality (hazard ratios of 7.49 and 6.27, respectively; *p* < 0.001). Interestingly, LMWH use was not associated with a decreased incidence of VTE when stratified by risk groups. **Conclusions:** this study suggests that COVID-19 patients with high thrombosis and bleeding risk scores have a higher mortality rate.

## 1. Introduction

Coronavirus disease 2019, or COVID-19, is an emerging infectious respiratory disease that has spread globally. The number of cases requiring medical care has markedly strained hospitals and healthcare resources, including isolation wards, intensive care unit beds, ventilators, and medical personnel. The virus primarily spreads person-to-person through respiratory droplets. The symptoms vary from mild (fever, cough, loss of taste/smell, fatigue, sore throat, headache, body aches, diarrhea, and rash) to severe pneumonia, with respiratory failure as the acute respiratory syndrome caused by coronavirus-2 (SARS-CoV-2). The associated complications of COVID-19 include venous thromboembolism (VTE), superimposed infection, systemic inflammation, and death.

Several studies have demonstrated a high incidence of VTE (pulmonary embolism) as well as arterial thrombosis such as stroke. COVID-19 itself can lead to a hypercoagulable state due to excessive inflammation, immobilization, and disseminated intravascular coagulopathy (DIC) [1,2]. Scientific organizations have suggested anticoagulation to prevent thromboembolic events among severe or critically hospitalized COVID-19 patients without contraindications [1,3]. A prospective study showed that 5.7% of hospitalized COVID-19 patients receiving intermediate or therapeutic doses of anticoagulants for VTE prophylaxis experienced major bleeding, and 6.7% had non-major bleeding [4]. The patients with major bleeding had a more than 2-fold higher mortality rate than those who did not bleed. The decision to prescribe anticoagulants for critically ill patients should be based on both guidelines and local research, as clinical and healthcare situations vary among different countries. Application of the risk assessment models (RAMs) to calculate the risk of VTE and bleeding can guide thromboprophylaxis, avoiding overuse of anticoagulants, which increase the risk of bleeding [5]. The candidate VTE-RAMs to detect VTE among COVID-19 patients are the Padua prediction score (PPS) and International Medical Prevention Registry (IMPROVE) RAM to detect a VTE risk stratification [6]. The counterpoint of IMPROVE also predicts bleeding risk among sepsis patients. Newer approaches have modified the RAM for predicting COVID-19 complications, but evidence of the efficacy of RAM scores in Thai patients are lacking. Anticoagulant prophylaxis is not uniformly given in Thailand.

The decision to use anticoagulants should be carefully considered by healthcare professionals, weighing the potential benefits of preventing VTE events against the bleeding risk. Therefore, both risk scores need to be evaluated. Thus far, there has been little knowledge in evaluating thrombosis against bleeding risks and comparing patients with or without VTE prophylaxis in COVID-19 infections. The objectives of the present study were to assess the relationship between thrombosis/bleeding risk score and the rates of thrombosis, bleeding, and 30-day mortality among patients with COVID-19 infections. These outcomes were also correlated with the use of low molecular weight heparin (LMWH) prophylaxis, per the decisions of the attending physicians.

## 2. Materials and Methods

### 2.1. Study Population and Definition 

A retrospective cohort study reviewed the archived medical records of adult patients aged ≥ 18 years with COVID-19 infections. They were admitted to either the Rajavithi or the Rangsit-Rajavithi Hospitals, which were tertiary care medical centers from 2021 to 2022. Laboratory confirmation of COVID-19 infection was performed using a polymerase chain reaction (PCR) assay of nasal or pharyngeal swab specimens. This study was approved by the Ethics Committee of the Rajavithi Hospital (Number 198/2564).

### 2.2. Data Analysis 

Classification of the severity of COVID-19 was categorized as follows. “Moderate” meant individuals presenting with evidence of lower respiratory disease during clinical assessment or imaging and who had an oxygen saturation measured using pulse oximetry (SpO_2_) of ≥94% of room air. Severely ill patients had either an SpO_2_ < 94%, respiration > 30 breaths/min, or lung infiltrates in more than 50% of their chest film [7]. Critically ill patients had respiratory failure, septic shock, and/or multiple organ dysfunction.

The Padua prediction score of VTE (PPS_VTE_) [8,9,10] was calculated for each patient using the following criteria: active cancer (3 points), previous VTE (3 points), reduced mobility (3 points), already known thrombophilia condition (3 points), recent (within one month) trauma or surgery (2 points), age (≥70 years) (1 point), heart and/or respiratory failure (1 point), acute myocardial infarction or ischemic stroke (1 point), acute infection and/or rheumatologic disorder (1 point), ongoing hormonal therapy (1 point), and body mass index (BMI) ≥ 30 kg/m^2^ (1 point). A cut point of a high risk of VTE was a cumulative PPS_VTE_ of ≥4, and the remainder were classified as low risk.

The IMPROVE risk score of VTE (IMPROVE_VTE_) [11,12,13,14] summed the following risk factors: previous VTE (3 points), known thrombophilia (2 points), current lower-limb paralysis (2 points), current cancer (2 points), immobilized more than a week (1 point), admitted to intensive care unit or equivalent (1 point), and age > 60 years (1 point). IMPROVE_VTE_ scores of ≥4, 2–3, and 0–1 were categorized as high, moderate, and low VTE risk, respectively. The IMPROVEDD score of VTE (IMPROVEDD_VTE_) included immobilization, ICU/CCU stay, and an age above 59 years (1 point each), known thrombophilia, current limb paralysis, cancer, D-dimer levels 2 times above or below the normal limit (2 points each), and previous VTE (3 points). A high risk of thrombosis was defined as a cumulative IMPROVEDD_VTE_ ≥ 2, and a low risk was defined as 0–1 points [15]. 

The IMPROVE bleeding risk score (IMPROVE_BRS_) was calculated by adding the occurrence of an active gastroduodenal ulcer (4.5 points), bleeding event within the past 3 months (4 points), platelet count at admission < 50 × 10^9^ cells/L (4 points), hepatic failure (2.5 points), ICU/CCU stay (2.5 points), central venous catheter insertion (2 points), active rheumatic disease (2 points), active malignancy (2 points), age of 40–80 years (1.5 points), age of ≥85 years (3.5 points), estimated glomerular filtration rate (eGFR) 30–59 mL/min (1 point), and eGFR < 30 mL/min (2.5 points) [16]. A high risk of bleeding was defined as a cumulative IMPROVE_BRS_ score of ≥7, and a low risk was defined as a score of <7.

Events of interest included major bleeding or thrombosis events occurring within 30 days after receiving a positive COVID-19 test. Major bleeds were defined as bleeds that were fatal, involving a critical site, leading to a hemoglobin drop of at least 2 g/dL, or requiring a packed red cell transfusion of ≥2 units/day [17]. If the patients had clinical suspicion of thrombosis, the physicians considered performing CT pulmonary angiography (CTPA) or Doppler ultrasound of extremities as appropriate [18]. The primary end point of the study was death within 30 days of hospitalization.

### 2.3. Statistical Analysis

Categorical variables were presented as numbers and percentages. Continuous variables were presented as means with standard deviations for normally distributed data, and as medians with interquartile ranges (IQR) for non-normally distributed data. The Kaplan–Meier method was used for the survival analysis, and the Cox proportional hazard model was used to relate the risk factors to the survival time. The area under the receiver operating characteristic (ROC) curve (AUC) was determined to estimate the AUC of VTE or bleeding events to predict the 30-day mortality. All the data were analyzed using SPSS program version 22.0 (Mahidol license). *p*-values of ≤0.05 were considered statistically significant.

## 3. Results

### 3.1. Patient Characteristics

The cohort comprised 160 patients with COVID-19 infections admitted to either the Rajavithi or the Rangsit-Rajavithi Hospitals. The median age was 59.0 years (IQR 46.0–69.0), with 69.4% being female. Metabolic disorders included hypertension (77.9%), dyslipidemia (33.1%), and type 2 diabetes mellitus (27.5%). The disease severities were moderate (41.3%; *n* = 66) and severe (40.0%; *n* = 64). The most common clinical presentations were fever (91.3%), cough (78.1%), and dyspnea (73.1%). The ICU admission rate was 25.6%. Fifty-three of the patients received a prophylaxis dose, and twenty of the patients received a therapeutic dose of anticoagulant. The median length of hospital stay was 15.0 days (range: 1–60). Fifty-eight of the patients (36.3%) died during hospitalization. The most common causes of death were bacterial infection (91.4%), cardiovascular system failure (3.5%), bleeding, active malignancy, and venous thromboembolism (1.7% each) (Table 1). Complications were found in 73.8% of the cases, such as superimposed infections, electrolyte imbalances, and hyperglycemic conditions. 

### 3.2. Thrombosis and Bleeding Prognostic Scores Could Predict Thrombosis and Bleeding

The criteria of PPS_VTE_, IMPROVE_VTE_, and IMPROVE_BRS_ were stratified according to the details shown in the Materials and Methods Section 2. Twenty (12.5%) of the patients developed thrombosis, while the mortality rate was 36.3%. A high PPS_VTE_ was associated with thrombosis, with event rates of 8 (30.8%) versus 12 (9.0%) for high and low scores, respectively (*p* = 0.006). Additionally, 11 (25.6%) vs. 9 (7.7%) VTE events occurred in patients with high vs. low IMPROVE_VTE_ risk scores, respectively (*p* = 0.002). 

Thirty-seven of the patients (23.1%) experienced bleeding during hospitalization. Hemorrhage occurred in 29.0% vs. 72.2% of the patients with low vs. high IMPROVE_BRS_ scores, respectively (*p* = 0.001). There was no significant association between LMWH and major bleeding. 

### 3.3. Thrombosis and Bleeding Prognostic Scores Could Predict Mortality

A high PPS_VTE_ was associated with a high mortality, showing a hazard ratio (HR) of 2.80 (95% confidence interval [CI], 1.09–7.13). The HRs for high IMPROVE_VTE_, high IMPROVEDD_VTE_, and high IMPROVE_BRS_ scores were 7.49 (95% CI, 3.82–14.67), 5.83 (95% CI, 3.26–10.44), and 6.27 (95% CI, 3.60–10.91), respectively (Table 2). 

The combined thrombosis and bleeding risk assessment models showed higher HRs. The HR of the combination of high PPS_VTE_ and high IMPROVE_BRS_ scores (group 1) was 7.40; 95% CI, 3.18–14.48 (*p* < 0.001). The combined high IMPROVE_VTE_ + high IMPROVE_BRS_ (group 2) and high IMPROVEDD_VTE_ + high IMPROVE_BRS_ scores (group 3) similarly revealed HRs of 6.29 (95% CI, 3.54–11.16 (*p* < 0.001)), as shown in Table 3. The predicting model of death achieved a high area under the receiver operator characteristic curve (AUC-ROC) of 0.60 (95% CI, 0.50–0.69; *p* = 0.03) in group 1 and 0.66 (95% CI 0.56–0.74; *p* = 0.001) in groups 2 and 3. However, the model did not demonstrate statistical significance in forecasting thrombosis and bleeding events among the patients with COVID-19 infections. 

A subgroup analysis for predicting death associated with thrombosis (*n* = 14) was performed based on three models: PPS_VTE_, IMPROVE_VTE_, and IMPROVEDD_VTE_. The AUC-ROC for death associated with thrombosis was 0.834 (95% CI, 0.74–0.92; *p* < 0.001) for patients with a high IMPROVEDD_VTE_ score, 0.744 (95% CI, 0.60–0.88; *p* = 0.003) for patients with a high IMPROVE_VTE_ score, and 0.68 (95% CI, 0.51–0.84, *p* = 0.028) for patients with a high PPS_VTE_ score of ≥4.

**Figure 1:**
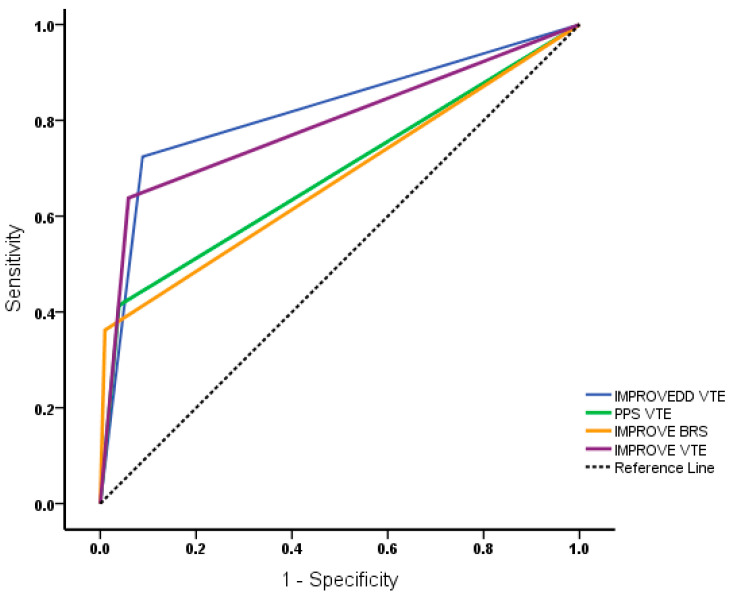
Receiver operating characteristic (ROC) curves of the venous thromboembolism (PPS_VTE_, IMPROVE_VTE)_ and bleeding (IMPROVE_BRS)_ risk score models for mortality prediction. The areas under the curves (AUC) were 0.68 (PPS_VTE_), 0.79 (IMPROVE_VTE_), 0.67 (IMPROVE_BRS_), and 0.82 (IMPROVEDD_VTE_).

**Figure 2:**
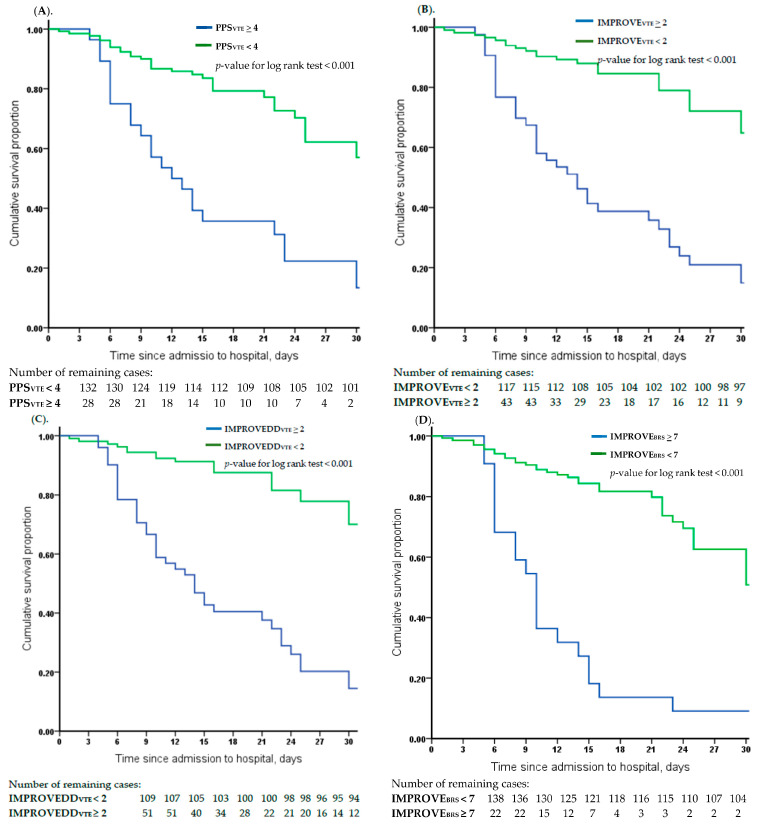

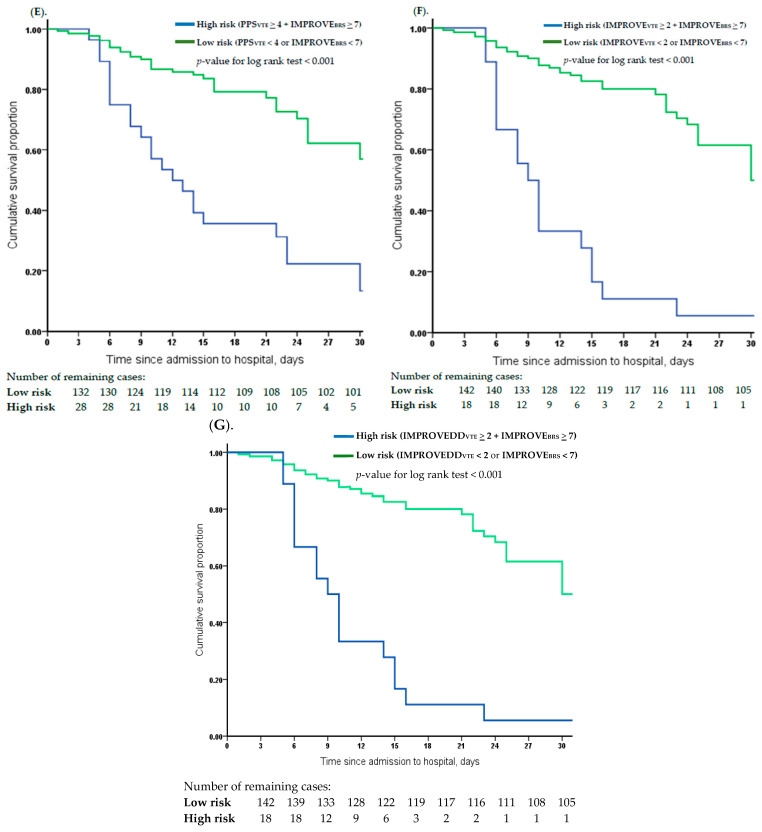
Kaplan–Meier curve for cumulative survival according to difference venous thromboembolism and bleeding risk scores among 160 patients. (**A**) Comparison of cumulative survival rates between a Padual prediction score of VTE of <4 and ≥4 subgroup; *p*-value < 0.001. (**B**) Comparison of cumulative survival rates between an IMPROVE prediction score of VTE of <2 and ≥2 subgroup; *p*-value < 0.001. (**C**) Comparison of cumulative survival rates between an IMPROVEDD prediction score of VTE of <2 and ≥2 subgroup; *p*-value < 0.001. (**D**) Comparison of cumulative survival rates between an IMPROVE prediction score of bleeding of <7 and ≥7 subgroup; *p*-value < 0.001. (**E**) Comparison of cumulative survival rates between a combination of PPS_VTE_ (≥4) + IMPROVE_BRS_ (≥7) (high risk) and a combination of PPS_VTE_ (<4) or IMPROVE_BRS_ (<7) (low risk) prediction score subgroups; *p*-value < 0.001. (**F**) Comparison of cumulative survival rates between a combination of IMPROVE_VTE_ (≥2) + IMPROVE_BRS_ (≥7) (high risk) and a combination of IMPROVE_VTE_ (<2) or IMPROVE_BRS_ (<7) (low risk) prediction score subgroups; *p*-value < 0.001. (**G**) Comparison of cumulative survival rates between a combination of IMPROVEDD_VTE_ (≥2) + IMPROVE_BRS_ (≥7) (high risk) and a combination of IMPROVEDD_VTE_ (<2) or IMPROVE_BRS_ (<7) (low risk) prediction score subgroups; *p*-value < 0.001.

We subsequently compared the ROC curves to assess the accuracy of the VTE or bleeding RAM in predicting mortality rates. The AUC was 0.68 (95% CI, 0.59–0.77) for PPS_VTE_, 0.79 (95% CI, 0.70–0.87) for IMPROVE_VTE_, 0.67 (95% CI, 0.58–0.76) for IMPROVE_BRS_, and 0.82 (95% CI, 0.74–0.89) for IMPROVEDD_VTE_ (Figure 1). The survival analyses showed that PPS_VTE_, IMPROVE_VTE_, IMPROVEDD_VTE_, and IMPROVE_BRS_, as well as their combined scores, could significantly predict the 30-day mortality due to COVID19 infection (*p* < 0.001), as shown in Figure 2. 

### 3.4. Low Molecular Weight Heparin Prophylaxis and Outcomes

In this study, LMWH prophylaxis was given in 73 of the patients (45.6%). The thrombotic rates of the patients receiving low molecular weight heparin (LMWH) were not significantly different from the patients who did not receive LMWH. The HR was 1.96 (95% CI, 0.24–16.12) in the high PPS_VTE_, 2.51 (95% CI, 0.52–12.01) in the moderate-to-high IMPROVE_VTE_, 2.16 (95% CI, 0.59–7.82) in the high IMPROVEDD_VTE_, and 0.79 (95% CI, 0.19–3.21) in the high IMPROVE_BRS_ subgroups (Table 4). Among the 53 patients who received LMWH as thromboprophylaxis, 47.2% survived their hospitalization for COVID-19. Interestingly, the analysis identified statistically significant differences between the survivors and non-survivors. The patients of an older age (*p* = 0.005) and those requiring intensive care unit (ICU) admissions (*p* < 0.001) had higher mortality rates.

**Figure 3:**
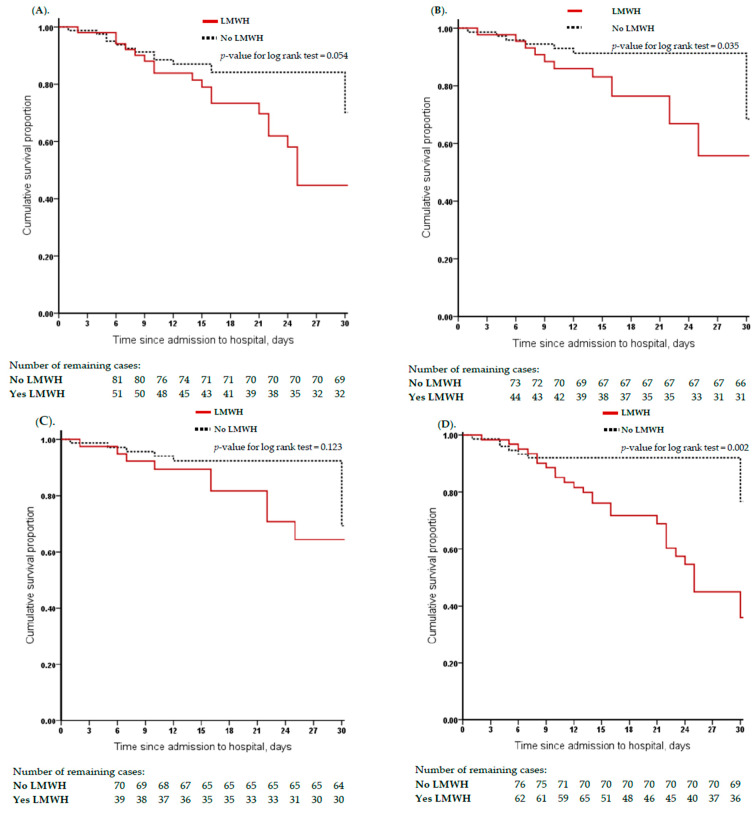

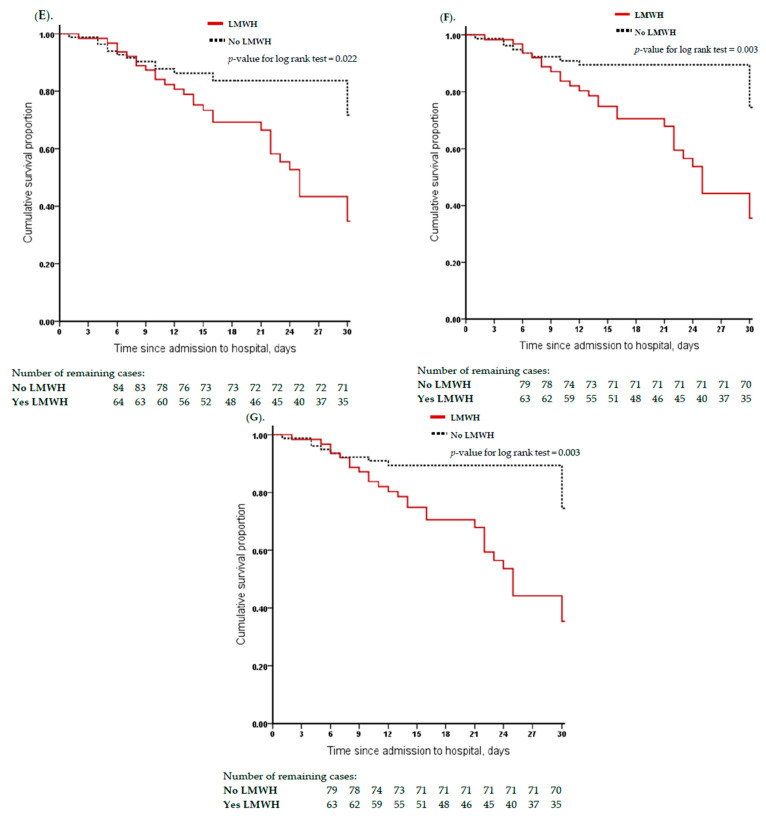
Kaplan–Meier curve for cumulative survival rates between patients with and without low molecular weight heparin, according to each low venous thromboembolism and bleeding risk score. (**A**) Comparison of cumulative survival rates among patients with Padual prediction scores of VTE ≥ 4 subgroup; *p*-value = 0.054. (**B**) Comparison of cumulative survival rates among patients with IMPROVE prediction scores of VTE ≥ 2; *p*-value = 0.035. (**C**) Comparison of cumulative survival rates among patients with IMPROVEDD prediction scores of VTE ≥ 2; *p*-value = 0.123. (**D**) Comparison of cumulative survival rates among patients with IMPROVE prediction scores of bleeding ≥ 7; *p*-value = 0.002. (**E**) Comparison of cumulative survival rates among patients with PPS_VTE_ (≥4) + IMPROVE_BRS_ (≥7) (high risk) prediction score subgroup; *p*-value = 0.022. (**F**) Comparison of cumulative survival rates among patients with IMPROVE_VTE_ (≥2) + IMPROVE_BRS_ (≥7) (high risk) prediction score subgroup; *p*-value < 0.003. (**G**) Comparison of cumulative survival rates among patients with IMPROVEDD_VTE_ (≥2) + IMPROVE_BRS_ (≥7) (high risk) prediction score subgroup; *p*-value < 0.003.

In the high-risk score groups, there was no difference in survival between the LMWH vs. no LMWH groups (Table 2). However, in the low IMPROVE_VTE_ and IMPROVE_BRS_ risk subgroups, the patients who received LMWH showed significantly higher mortality rates (Figure 3B,D–G).

## 4. Discussion 

This study found that combined thrombosis and bleeding predicting scores were strongly associated with 30-day mortality in Thai hospitalized patients with COVID-19 infections. Specifically, the presence of high PPS_VTE_, IMPROVE_VTE_, IMPROVEDD_VTE_, and IMPROVE_BRS_ scores was correlated with high mortality rates. We also confirmed their predictive roles for thrombosis and bleeding based on previous studies. Increases in the 30-day mortality rate were also associated with LMWH usage, especially in the patients with low PPS_VTE_, IMPROVE_VTE_, and IMPROVE_BRS_ scores. These data did not support the roles of LMWH in improving COVID19 outcomes, especially in patients with low VTE risks. 

Although the treatment guidelines suggested that all hospitalized patients receive thromboprophylaxis, we could not demonstrate a reduction in VTE rates due to LMWH administration. The trend was towards an increase in VTE due to LMWH prophylaxis in each risk group, but there was no statistical significance. Our study did not detect an increase the bleeding risk due to LMWH administration. It is possible that the attending physicians were more likely to give LMWH to more severe patients with low bleeding risks based on their experience, although they were in the same risk score categories. Further strategies to decrease VTE in these high-risk groups are required. 

The majority of the patients in our study were female, which differs from other studies [5,19]. For male patients with COVID-19 infection, both clinical severity and VTE events are higher [20]. The higher rate of mortality in men may be explained by several factors; an association between sex differences in the immune system concluded that sex-based immunological differences contribute to variations in susceptibility to infection. A study by Bienvenu et. al. postulated that angiotensin-converting enzyme 2 (ACE2) expression is higher in males, potentially facilitating increased viral entry and contributing to worse outcomes [21]. Women, especially during their reproduction years, are at an increased risk of autoimmune disease but are more resistant to infection than men [22,23]. Metabolic disorders, including hypertension, dyslipidemia, and type 2 diabetes mellitus, were the most common comorbidities in the current study. The analysis revealed an increasing trend of mortality of 1.6 times among diabetic patients (*p* = 0.06). However, the other metabolic disorders, including hypertension (*p* = 0.411) and dyslipidemia (*p* = 0.383), did not show any statistically significant differences in mortality rates between the patients with and without those conditions.

Thromboprophylaxis guidelines for patients with COVID-19 infection recommend that all hospitalized patients receive a pharmacologic dose of prophylaxis [24]. However, hospitalized patients with COVID-19 infection should be assessed based on their VTE risk using risk assessment models (Padua, IMPROVE) to guide thromboprophylaxis, because VTE prophylaxis is not necessary for all patients. The current study showed that only 16.2% of the patients were in the high-risk group based on the Padua prediction score, which was lower than in previous studies [5,25]. Although more high-risk patients were found in previous studies, the incidence of developing VTE was only 11.0% [25] compared to the current study, which found 30.8% VTE. A higher VTE rate may be explained by longer hospitalizations and higher complications occurring during the course of disease, such as acute respiratory distress syndrome, systemic inflammatory response syndrome, shock, and multiorgan failure. Direct involvement of the virus with affected the endothelial cells caused severe endothelial injury, endothelitis, increased angiogenesis, and widespread vascular thrombosis with microangiopathy and occlusion of alveolar capillaries. 

Patients with a low VTE risk also had low COVID-19 mortalities and did not benefit from thromboprophylaxis. Evidence supporting the benefits LMWH prophylaxis is limited among patients with COVID-19 infections. Based on our findings, we believe that robotic or artificial intelligence (AI) could be particularly valuable tools in identifying patients who require pharmacological thromboprophylaxis [26]. This is especially relevant in the context of pandemics of contagious diseases, such as COVID-19, which placed a significant burden on healthcare personnel and led to overwhelming healthcare utilization [27]. Our study proposed the use of prediction scores not only for evaluating the risks of thrombosis and bleeding but also for predicting in-hospital mortality rates. This result was similar to those of other internal medicine patients [6,28]. This may be explained by immune thrombosis, which may play a role in the pathogenesis of the COVID-19 virus [29,30]. The benefits of LMWH might indicated among COVID-19 patients with high risk scores [31,32], but this needs to be proven through randomized controlled trial studies. However, it is important to exercise caution in the administration of anticoagulants, as their excessive use may increase the risk of adverse outcomes, including a higher 30-day mortality rate. Therefore, a balanced approach is required when managing patients who have both a predisposition to blood clot formation (thrombosis) and a tendency towards bleeding.

There were limitations of this study. Firstly, this was a retrospective study that was limited to patients who were hospitalized with COVID-19 infection in two institutions; therefore, it might not be representative of populations in different countries. Second, the decisions to give LMWH were made according to clinical judgement, not randomization. Third, we were unable to explore individual parameters for each risk factor model due to an insufficient sample size. Fourth, our study included only a small number of patients who received prophylactic LMWH. Therefore, we could not definitively conclude that LMWH was not associated with a reduced risk of VTE due to the lack of statistical power. Finally, the cause of death was determined clinically, not by an autopsy. Future studies including larger samples of COVID-19 patients are required to confirm the findings of this study.

## 5. Conclusions

Patients with high PPS_VTE_, IMPROVE_VTE_, and/or IMPROVE_BRS_ scores had a high risk of death. This group requires special attention and research to improve their outcomes. The risk score had a good performance for predicting death associated with thrombosis in patients with COVID-19 infections who have high IMPROVEDD_VTE_ levels above the thresholds. Patients with low PPS_VTE_, IMPROVE_VTE_, and IMPROVE_BRS_ risk scores were unlikely to be benefit from LMWH prophylaxis.

## Figures and Tables

**Table 1:** Characteristics of the study population (*n* = 160).

Characteristics	*n* (%)	
Age (years) **	59.0	(46.0–69.0)
Sex		
Female	111	(69.4)
BMI (kg/m^2^) *	27.51	±5.54
Co-morbidities	111	(69.4)
Hypertension	86	(77.9)
Dyslipidemia	53	(33.1)
Diabetes mellitus	44	(27.5)
Chronic kidney disease	13	(11.7)
Cerebrovascular disease	5	(4.5)
Atrial fibrillation	5	(4.5)
Clinical presentation		
Fever	146	(91.3)
Cough	125	(78.1)
Dyspnea	117	(73.1)
Runny nose	24	(15.0)
Sore throat	23	(14.4)
Diarrhea	18	(11.3)
Myalgia	13	(8.1)
Disease severity		
Moderate	66	(41.3)
Severe	64	(40.0)
Critical	30	(18.8)
Ward		
Non-ICU	119	(74.4)
ICU	41	(25.6)
Prophylaxis dose LMWH	53	(33.1)
Therapeutic dose LMWH	20	(12.5)
Length of stay (days) **	15	(1–60)
Death	58	(36.3)
Cause of death		
Bacterial infection	53	(91.4)
Cardiovascular	2	(3.5)
Bleeding	1	(1.7)
Venous thromboembolism	1	(1.7)
Cancer	1	(1.7)

Abbreviations: BMI, body mass index; ICU, intensive care unit; LMWH, low molecular weight heparin. * Mean ± SD, ** median (interquartile range).

**Table 2:** Hazard ratio of mortality according to thrombosis/bleeding risk assessment models among 160 patients with COVID19 infections.

Risk Score	Hazard Ratio	95% CI	*p*-Value
PPS_VTE_ *			
Low	1 (ref)		
High	2.80	1.09–7.13	0.031
IMPROVE_VTE_ *			
Low	1 (ref)		
Moderate	3.55	1.91–6.61	<0.001
High	7.49	3.82–14. 67	<0.001
IMPROVE_VTE_ *			
Low	1 (ref)		
Moderate to high	1.77	0.91–3.47	0.092
IMPROVEDD_VTE_ *			
Low	1 (ref)		
High	5.83	3.26–10.44	<0.001
IMPROVE_BRS_ **			
Low	1 (ref)		
High	6.27	3.60–10.91	<0.001

Abbreviations: CI, confidence interval. * The thrombosis events are as follows: all thrombosis, *n* = 20; pulmonary embolism, *n* = 6; myocardial infarction, *n* = 6; systemic embolism, *n* = 6; deep vein thrombosis, *n* = 1; ischemic stroke, *n* = 1. ** There were 47 bleeding events.

**Table 3:** Hazard ratios of mortality according to a combination of thrombosis/bleeding risk assessment models.

Risk Score	HR	95% CI	*p*-Value	Death			Thrombosis			Bleeding		
				AUC	95% CI	*p*-Value	AUC	95% CI	*p*-Value	AUC	95% CI	*p*-Value
All patients												
PPS_VTE_ ≥ 4 + IMPROVE_BRS_ ≥ 7 (*n* = 12)												
No	1 (ref)											
Yes	7.40	3.78–14.48	<0.001	0.60	0.50–0.69	0.03	0.57	0.42–0.71	0.302	0.55	0.44–0.67	0.335
IMPROVE_VTE_ ≥ 2 + IMPROVE_BRS_ ≥ 7 (*n* = 18)												
No	1 (ref)											
Yes	6.29	3.54–11.16	<0.001	0.66	0.56–0.74	0.001	0.60	0.46–0.75	0.122	0.56	0.44–0.68	0.264
IMPROVEDD_VTE_ ≥ 2 + IMPROVE_BRS_ ≥ 7 (*n* = 18)												
No	1 (ref)											
Yes	6.29	3.54–11.16	<0.001	0.66	0.56–0.74	0.001	0.60	0.46–0.75	0.122	0.56	0.44–0.68	0.264

Abbreviations: HR, hazard ratio; AUC, area under the receiver operator characteristic curve; CI, confidence interval.

**Table 4:** Hazard ratio of thrombosis/bleeding events among patients who were classified by thrombosis/bleeding risk scores and low molecular weight heparin usage.

Risk Score	Hazard Ratio	95% CI	*p*-Value	Risk Score	Hazard Ratio	95% CI	*p*-Value
High PPS_VTE_				Low PPS_VTE_			
Without LMWH	1 (ref)			Without LMWH	1 (ref)		
With LMWH	1.96	0.24–16.12	0.529	With LMWH	3.40	0.91–12.71	0.069
Moderate to high IMPROVE_VTE_				Low IMPROVE_VTE_			
Without LMWH	1 (ref)			Without LMWH	1 (ref)		
With LMWH	2.51	0.52–12.01	0.248	With LMWH	3.70	0.73–18.60	0.111
High IMPROVEDD_VTE_				Low IMPROVEDD_VTE_			
Without LMWH	1 (ref)			Without LMWH	1 (ref)		
With LMWH	2.16	0.59–7.82	0.239	With LMWH	3.71	0.37–36.33	0.260
High IMPROVE_BRS_				Low IMPROVE_BRS_			
Without LMWH	1 (ref)			Without LMWH	1 (ref)		
With LMWH	0.79	0.19–3.21	0.745	With LMWH	1.85	0.91–3.76	0.085

Abbreviations: CI, confidence interval.

## Data Availability

The original contributions presented in the study are included in the article material. Further inquiries can be directed to the corresponding author.
